# Microstructural and Textural Evolution of Cold-Drawn Mg–Gd Wires during Annealing Treatment

**DOI:** 10.3390/ma17030683

**Published:** 2024-01-31

**Authors:** Liuxia Sun, Jing Bai, Feng Xue, Kai Yan

**Affiliations:** 1 School of Arts and Sciences, Shanghai Dianji University, Shanghai 201306, China; 2 School of Materials Science and Engineering, Southeast University, Nanjing 211189, China; xuefeng@seu.edu.cn; 3 College of Mechanical Engineering, Yangzhou University, Yangzhou 225127, China; yankai@yzu.edu.cn

**Keywords:** Mg–Gd wire, annealing treatment, texture, microstructure

## Abstract

In addition to cold drawing, the process of annealing is also essential in the preparation of Mg-4.7 wt%Gd (G4.7) alloy wires. The effect of annealing treatment on the recrystallized microstructure and texture of cold-drawn G4.7 wires was investigated. The results demonstrate that the uniformity and regularity of the recrystallized grains, as well as the annealing texture, impact the follow-up cold drawing performance. When the as-drawn G4.7 wires were annealed at 375 °C, the recrystallized grains were refined, accompanied by uniformity and regularity. Accordingly, the G4.7 wire had a good subsequent drawing deformability, with a maximum accumulative true strain (ATS) of 144%. Additionally, the evolution of the microstructure was consistent with the evolution of the texture. While annealing at a lower temperature (325 °C), the {0002} basal texture of the G4.7 wire was weak, forming the main texture component <10$\bar{1}$0>//DD (the drawing direction). With the increase in temperature, the basal texture was gradually strengthened and the texture component transformed from <10$\bar{1}$0>//DD to a recrystallized texture based on <11$\bar{2}$0>//DD. Even under high-temperature annealing, the G4.7 wire was still affected by the cold-drawn deformation texture and could not fully recover to the as-extruded texture, thus causing a decrease in the subsequent drawing performance.

## 1. Introduction

Magnesium and its alloys are promising biomedical materials due to their high mechanical properties, complete biodegradability and good biocompatibility [1,2,3]. Among all the Mg products, Mg alloy wires that can be used to produce sutures, staples, woven stents for the digestive tract or blood vessels, etc., have shown more widespread application potential [4,5]. However, Mg alloys exhibit poor ductility and cold formability due to the hexagonal close-packed (hcp) structures. Micro-alloying with rare earth (RE) has been proven to be effective in weakening the basal texture or even in obtaining a non-basal texture; thus, it can enhance the room-temperature ductility and formability of Mg alloys [6,7]. In particular, the Gd element can greatly ameliorate the mechanical performances of Mg alloys, owing to the modification of the texture or aging process [8]. Furthermore, the Gd element has higher biosafety [9,10]; therefore, it is considered suitable as the main alloying element for preparing medical Mg alloy wires.

The cold drawing deformation of Mg–Gd alloy wires was elaborated on in our previous paper [11]. Nevertheless, in addition to the cold drawing process, the subsequent annealing process is an indispensable link during the preparation of Mg alloy wires. On the one hand, a new recrystallized microstructure of Mg alloys with refined grains is formed through annealing treatment, while eliminating work hardening during deformation. For example, Yang Ping et al. [12] effectively modified the deformation microstructure of Mg alloys with refinement grains and improved the rolling formability of Mg alloys through static recrystallization (SRX) annealing. On the other hand, annealing treatment can improve the texture of deformed Mg alloys, which is crucial for their subsequent plastic deformation. There have been many reports on the weakening of the texture in RE magnesium alloys during the SRX process [13,14,15,16]. It is reported that, upon annealing, SRX occurred in as-rolled Mg-1 wt.% (or 4 wt.%) Zn-1 wt.%Ce alloy and that the as-rolled basal texture was replaced with a weaker texture [13]. In addition, the study also showed that after 5 min of annealing, Mg-1Zn-1Ce had a lower basal texture, and as the annealing time extended to 60 min, the basal texture continued to decrease, while Mg-4Zn-1Ce exhibited a sudden decrease in texture intensity after 30 s of annealing, followed by a plateau trough and then an increase at 10 min [13]. Similarly, the significantly weakened texture was observed in a ZEK100 (Mg-1 wt.%Zn-0.4 wt.%Nd-0.3 wt.%Zr) alloy [14], a Mg-1 wt.% Zn-0.3~1.0 wt.%Ce alloy [15], and a Mg-2 wt.%Y alloy [16] during the SRX process. Therefore, SRX annealing can improve the microstructure of Mg alloy wires and weaken their texture; thus, it can contribute to the subsequent drawing of wires and to the improvement of the comprehensive mechanical properties of the wires.

The above studies mainly focus on the SRX of as-rolled Mg alloys, but there is currently little research on the SRX of cold-drawn Mg alloy wires [17,18]. In particular, there is even less research on the microstructural and textural evolution of cold-drawn Mg alloy wires containing the Gd element during annealing treatment [17]. Interestingly, Mg–Gd alloy wires experience some unique recrystallization behaviors after cold-drawn deformation followed by annealing; these behaviors are completely different from those of other deformation modes and alloying elements.

In addition to the deformation modes and alloying elements, there are many other factors that affect the SRX behavior of Mg alloy wires, such as texture, annealing temperature, and time, and thus affect the further drawing of the wires. In this research, various annealing treatments were performed at a temperature range from 325 to 475 °C and a time range from 5 to 120 min for cold-drawn Mg-4.7 wt% Gd (G4.7) wire to elucidate its microstructure and texture and their impact on mechanical properties. Furthermore, the SRX behavior of G4.7 during the annealing process can be clarified. In addition, this study also analyzes some factors that affect the further drawing performance, and it proposes the optimization of the annealing process and recrystallized microstructure. This is crucial for providing theoretical support for the design and manufacturing of Mg alloy wires and medical devices.

## 2. Materials and Methods

The preparation of Mg alloy wires mainly includes cold drawing and annealing treatment. The cold drawing deformation of G4.7 wires was detailed in our previous paper [11], and this study mainly involves the annealing process of G4.7 wires after cold drawing. Therefore, the composition of the experimental alloy is the same as that of the previous paper [11]. The experimental G4.7 ingot with a diameter of 60 mm was prepared by casting, followed by homogenization treatment at 530 °C for 20 h. The homogenized ingot then was hot extruded at 450 °C with an extrusion ratio of ~20. After that, as-extruded G4.7 wire with a thicker initial diameter of 3.0 mm was obtained. Starting from the as-extruded wires, the multi-pass cold drawing was performed step by step. The true strain of the first two passes was about ~3%; then, the true strain of the single pass was about ~7%. The thick wires were successively cold drawn with a maximum accumulative true strain (ATS) of ~165%, where the fracture occurred frequently (>50%).

With the aim of investigating the recrystallized microstructure and texture of the as-drawn Mg alloy wires, the G4.7 samples which followed the cold drawing underwent an annealing treatment in a radiation furnace with a relatively wide temperature range from 325 to 475 °C and a time range from 5 to 120 min.

To reveal the microstructure, optical microstructure (OM) analysis was performed on the G4.7 experimental wires with different annealing parameters. The metallographic characterization was carried out via an Olympus BX60M optical metallographic microscope (Olympus, Tokyo, Japan). According to the ASTM E112-96 (2004) standard [19], the metallographic photos of the central part of the wires were determined and counted using Image J software (Version 1.38) through the lineal intercept procedure, and there were more than 200 statistical intercepts for every specimen; thus, the information on the grain size and distribution of the Mg alloy wires was obtained. Additionally, to obtain a Tecnai G2 transmission electron microscope (TEM) (FEI, Eindhoven, The Netherlands) image of the G4.7 wire with 165% ATS, the specimen was thinned by twin-jet electropolishing in a solution of 5 mL of perchloric acid and 95 mL of ethanol. The annealing microstructures were further studied by electron back-scattered diffraction (EBSD), operating at 20 kV. For EBSD analysis, the specimens were mechanically ground and electropolished in a solution of 5% nital acid in ethanol at 15–20 V.

The microhardness of the samples was measured by an FM-700 microhardness tester (Future-Tech, Kawasaki, Japan) with a load of 300 g and a dwell time of 10 s. The average microhardness values were calculated from ten indentations. Uniaxial tension tests were performed along the drawing direction (DD) to measure the mechanical properties of annealed G4.7 alloy wires at room temperature (RT). Tensile samples of cylindrical wire with a gauge length of 10 cm were prepared and tested by a CMT5105 electronic universal testing machine (Sans, Shenzhen, China). Three to five specimens were tested for each measurement.

## 3. Results

### 3.1. Microstructures and Mechanical Properties

**Figure 1:**
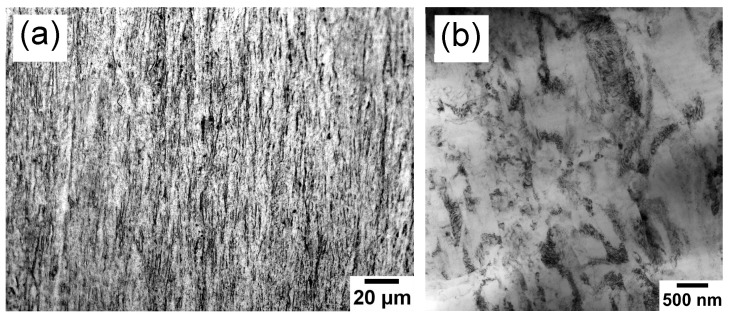
OM (**a**) and TEM (**b**) micrographs of as-drawn G4.7 wire with ATS of 165%.

Taking the sample state after cold drawing as the initial state, the initial OM microstructure of the G4.7 wire with 165% ATS is shown in Figure 1a; it is mainly composed of the fibrous structures along the DD without any visible equiaxed grain. The initial TEM photo of the cold-drawn G4.7 is shown in Figure 1b. It can be seen that there is a high dislocation density in the deformation microstructure of the wire with 165% ATS, indicating that the significant residual stresses were generated after cold drawing.

**Figure 2:**
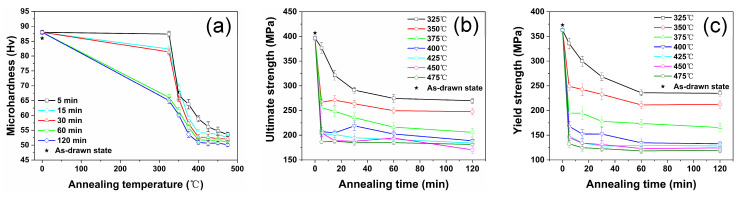
Mechanical property curves of the experimental wires, showing annealing treatment on (**a**) the microhardness, (**b**) the ultimate strength, and (**c**) the yield strength.

In addition to cold drawing, the process of annealing is also essential in the preparation of G4.7 alloy wires. To comprehensively explore the influence of the annealing process on the cold-drawn G4.7 alloy, the microhardness of the wire under different annealing treatments was measured, as shown in Figure 2a. Firstly, it can be observed that the annealing curves for 15 and 30 min are similar, while the annealing curves for 60 and 120 min are similar. Secondly, the effect of annealing temperature on hardness is much more significant compared to that of time. Furthermore, all the curves show a similar changing trend, with the following typical stages: (1) the hardness decreases slowly, and the decrease value is small when annealed below 350 °C; (2) there is a sharp decline at higher temperatures (350~400 °C), resulting in rapid softening of the wire; and (3) there is a relatively stable region from 400 to 475 °C. In this temperature range, the hardness basically reaches equilibrium and remains at Hv 50–55, which is similar to the microhardness of the as-extruded G4.7 wire (~Hv 55) [11].

The room-temperature ultimate strength and the yield strength for the as-annealed G4.7 wires in tension are presented in Figure 2b and Figure 2c, respectively. The ultimate strength and yield strength both decrease with the increase in annealing temperature until reaching equilibrium, showing the same trend. After annealing at 475 °C/120 min, the ultimate strength and yield strength are 181 and 119 MPa, respectively. Compared to the as-drawn G4.7 with ATS 165%, they decrease by 53% and 65%, and the strength is greatly reduced. Additionally, it shows more clearly that the effect of annealing temperature on strength is more significant compared to that of time.

**Figure 3:**
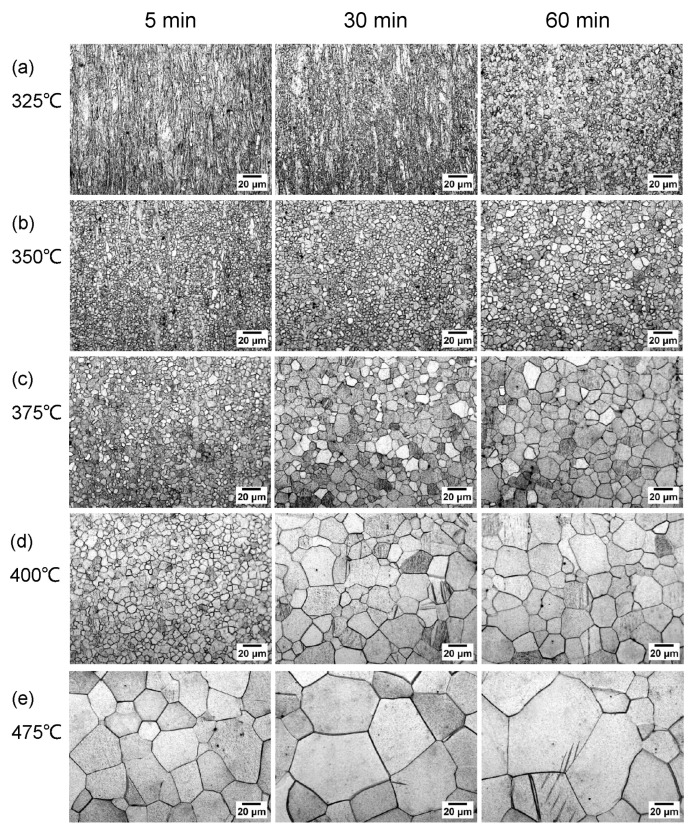
Optical micrographs of 165% drawn G4.7 in the subsequent further annealing process at different temperatures for 5 min, 30 min, 60 min: (**a**) 325 °C, (**b**) 350 °C, (**c**) 375 °C, (**d**) 400 °C, (**e**) 475 °C.

Metallographic observation was conducted on all the as-annealed G4.7 wires, and it could be found that the evolution of the microstructure fully demonstrated the changes in the mechanical properties. Based on the previous experimental results, some representative metallographic micrographs are shown in Figure 3, where only the annealed microstructures with a temperature range from 325 to 400 °C and the highest temperature of 475 °C after 5, 30, 60 min were chosen for comparison.

From Figure 3a, it can be seen that there is no significant change in the deformed microstructure of the G4.7 wire after 325 °C/5 min annealing, while the fibrous structure along the DD decreases after 30 min. Eventually, the fibrous structure disappears after 60 min of annealing. Although many small recrystallized grains are formed with a size of ~3 μm, the recrystallization is still incomplete. The microstructure of the as-annealed wire at 350 °C is indicated in Figure 3b. After 30 min, the microstructure still exhibits incomplete recrystallization, but the recrystallization ratio has increased compared to the wire annealed for 5 min. Complete recrystallization occurs after 60 min of annealing, but the microstructure is uneven with a grain size of approximately 4.8 μm. In contrast, a complete SRX occurs at 375 °C after annealing for 30 and 60 min (Figure 3c), in which the equiaxed grain is uniformly distributed and the average grain size is 9.4 and 12.3 μm, respectively. At a higher annealing temperature of 400 °C (Figure 3d), the microstructure completely recrystallizes after only 5 min, along with an average grain size of ~6.8 μm, and the grains gradually grow as the annealing time is prolonged. When annealed at higher temperatures (425, 450 °C), the grains in the G4.7 microstructure continue to grow; to condense the article, this microstructure is not listed here. At the highest annealing temperature of 475 °C for only 5 min, the recrystallized grains are already very large, ~29.8 μm. As the annealing time is prolonged, the grains continue to grow, and the large grains are mixed with the small grains, resulting in uneven distribution of the microstructure.

In order to quantify the recrystallized grain size and its uniformity, over 200 intercepts were calculated using a linear intercept method. Based on the results of the above microstructures and mechanical properties, the G4.7 wires annealed at three suitable temperatures were selected to continue the cold drawing experiment. The important statistics of the as-annealed G4.7 wires and the maximum ATS after annealing are shown in Table 1. According to the statistical results, the CV value of the grains annealed at 375 °C is significantly smaller than those annealed at 350 and 400 °C, suggesting that the grain size distribution is more uniform during 375 °C annealing. The recrystallized microstructure further affects the follow-up cold drawing process. From Table 1, we note that the ATS of the 350 °C annealed G4.7 is only ~131%, which is lower than that of the wire annealed at a higher temperature, indicating that the regularity and uniformity of the microstructure have a significant impact on the subsequent drawing deformability. Nevertheless, it should be noted that the drawing deformability of the annealed wire is lower than that of the as-extruded wire, despite the fact that the grain size of the 375 °C annealed wire is significantly smaller than that of the as-extruded wire (~165%), indicating that besides the grain refinement and microstructural uniformity, there are many other factors that affect the plastic deformability of Mg alloy wire, such as annealing texture.

### 3.2. Textural Examinations

By studying the microstructure and mechanical properties of G4.7 wire under different annealing treatments, the texture of G4.7 wire at different stages of recrystallization (the starting recrystallization, complete recrystallization, formation of larger equiaxed grains, and significant grain growth) was investigated.

**Figure 4:**
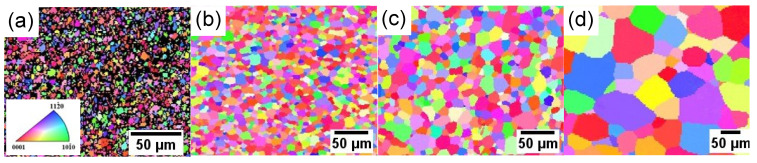
The EBSD orientation maps showing microstructure of as-annealed G4.7 wire: (**a**) 325 °C/60 min, (**b**) 375 °C/30 min, (**c**) 400 °C/30 min, (**d**) 475 °C/120 min.

Figure 4a–d depict the EBSD orientation maps of the G4.7 wires annealed at 325 °C/60 min, 375 °C/30 min, 400 °C/30 min, and 475 °C/120 min, corresponding to the four different stages of recrystallization mentioned above, respectively. After annealing at 325 °C/60 min, the G4.7 wire undergoes an incomplete recrystallization, and there is still a deformed microstructure, as well as a large number of irregularly shaped grains and wavy grain boundaries. Additionally, it is found that there are black areas in Figure 4a, which is due to the relatively high residual stress during the incomplete recrystallization stage, resulting in a lower confidence index and resolution. The annealing temperature (375 °C) is raised to achieve complete recrystallization and uniform grain distribution. As the annealing temperature further increases, the grains grow significantly, as shown in Figure 4c,d.

**Figure 5:**
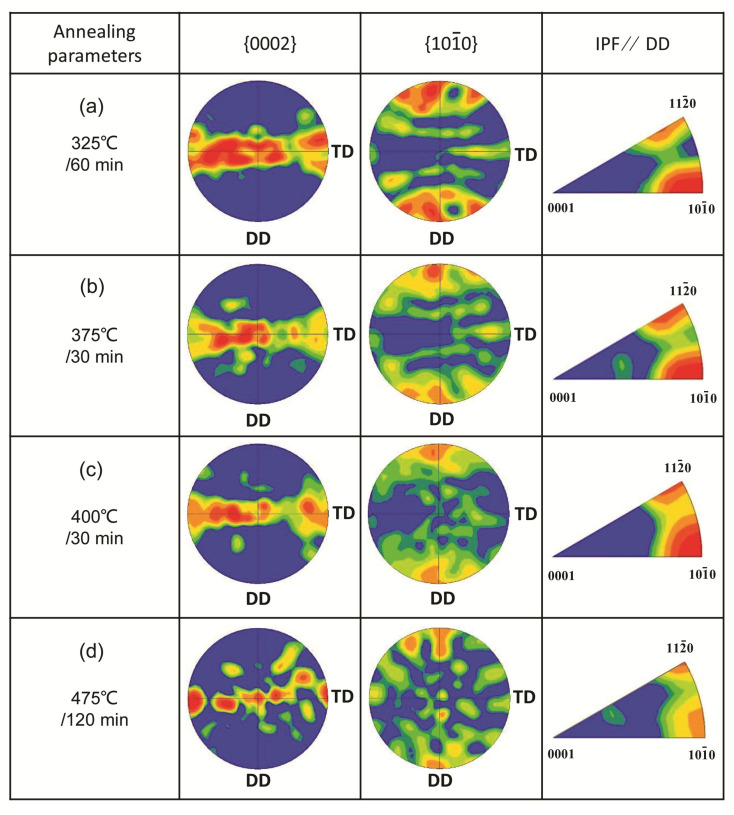
Textural evolution showing PF and IPF of G4.7 wire under different annealing treatments.

Figure 5a–d show the pole figures (PF), including {0002} and {10$\bar{1}$0}, and the inverse pole figures (IPF) parallel to the DD that correspond to Figure 4a–d, respectively. After annealing at 325 °C/60 min (Figure 5a), the {0002} basal texture for the G4.7 wire is weak, forming the main texture component <10$\bar{1}$0>//DD. As SRX proceeds (Figure 5b,c), the basal texture component is significantly enhanced, but it deviates to a certain angle towards the transverse direction (TD). With the increase in temperature, the texture component transforms from <10$\bar{1}$0>//DD to a recrystallized texture based on <11$\bar{2}$0>//DD, as shown in the IPF. Moreover, analyzing the strength of the {0002} poles in Figure 5a–d, the pole density is 3.163, 4.242, 5.809, and 11.635 multiples of the random distribution (m.r.d.), respectively, indicating an increase in texture intensity. In summary, with the increase in annealing temperature, the basal texture of the G4.7 wires was gradually strengthened during the SRX process, while the texture component transformed from <10$\bar{1}$0>//DD to <11$\bar{2}$0>//DD. Thus, it is clear that the evolution of the texture is consistent with the evolution of the microstructure.

## 4. Discussion

### 4.1. Annealed Microstructures

The annealing treatment is an essential part of the preparation of Mg alloy wire. SRX can refine grains and improve the microstructure of Mg alloy wires, which facilitates the subsequent cold drawing deformation. The annealed microstructure of G4.7 wire is significantly different from that of pure Mg, indicating that they exhibit different SRX behaviors [18]. This may be related to their different cold-drawn deformation history, internal intermetallic compound phases, alloy elements, etc.

Based on the annealing microstructure and corresponding mechanical property data of G4.7 wire, it can be inferred that the recrystallization temperature of G4.7 wire is 350 °C, which is obviously higher than that of pure Mg and AZ31 alloys [18,20]. Such results were also found in the DRX of other different processes for Mg alloys containing Gd [21,22]. Considering pure Mg and the nearly single-phase microstructure of AZ31, the solute-driven effect should be an important factor affecting the SRX process. Generally, trace solute atoms can significantly increase the recrystallization temperature of the Mg alloy. Here, the microstructure of the G4.7 wire contained a small amount of solute precipitation. Moreover, the atomic radius of Gd is significantly larger than that of Al and Zn, and the interaction between the Gd solute and the recrystallized grain boundaries is stronger. In summary, the reason why the SRX temperature of the G4.7 was so high mainly depended on the strong interaction between the Gd solute and the grain boundaries, which suppressed the boundary mobility and made the recrystallization process more difficult.

**Figure 6:**
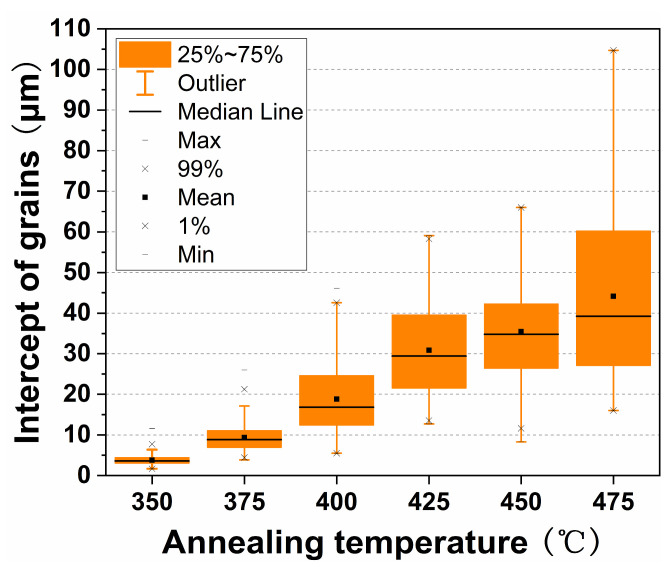
The grain intercept distribution of as-annealed G4.7 wire.

In addition, the influence of the as-annealed microstructure on the subsequent cold-drawn deformability is mainly reflected in the following aspects: the grain sizes and their uniformity and regularity. Through severe plastic deformation, some processing techniques (e.g., reciprocating extrusion [23] and high-pressure torsion [24]) can significantly refine grains. In this work, fine grain microstructures could also be obtained through cold drawing at room temperature and the subsequent annealing treatment. Based on the microstructure of the G4.7 wire, the distribution of the recrystallized grain size after annealing at different temperatures for 30 min was statistically analyzed, as shown in Figure 6. It can be seen that the grain size of the G4.7 gradually increased with the increasing annealing temperature. After annealing at 350~400 °C for 30 min, the recrystallized grain size of the G4.7 was lower than that of the as-extruded G4.7 (~21 μm). With the continuing temperature increase, the grains grew rapidly and the size was larger than that of the as-extruded wire. Additionally, after annealing at higher temperatures, the uniformity of the recrystallized grains was relatively poor; this can be seen in Figure 6, where the distribution of the grain size is very wide. For example, after annealing at 475 °C, the average grain size was ~44 μm, with a minimum grain size of ~16 μm and a maximum grain size of ~105 μm. By this point, the small grains were distributed around the large grains, which are also clearly visible in the metallographic photos. On the other hand, despite the fact that the grains were refined after annealing at a lower temperature (350 °C/30 min), the uniformity and regularity of the grains were poor due to incomplete recrystallization of the wires at this point. These inhomogeneous and disequilibrium grains resulted in a drawing performance, which led to premature crack initiation during a large deformation of the wires. Therefore, the cold drawing deformability of the G4.7 wire annealed at 350 °C was significantly lower than that of the as-extruded wire (Table 1). In contrast, as the temperature increased to 375 °C, the CV value decreased to 0.34 and a complete SRX occurred. Thus, the improvement of the uniformity and regularity of the recrystallized grains contributed to a better subsequent cold deformation, despite the coarser grains.

Simultaneously, it should be pointed out that the maximum ATS of the as-annealed G4.7 wire was still lower than that of the extruded wire, although the G4.7 wire exhibited a good deformability after annealing (as shown in Table 1). Therefore, in addition to the influence of grain size or intermetallic particles, there are other factors that have a significant impact on the cold drawing performance of as-annealed G4.7 wire, such as annealing texture.

### 4.2. Textural Analysis

Deformed Mg and its alloys undergo SRX during the annealing process, resulting in changes in the texture components and the formation of annealing texture. The formed annealing texture is closely related to the microstructure and is influenced by various factors, such as deformation texture, the alloying element, annealing temperature, and time [25]. When annealed at a lower temperature (325 °C/60 min), the G4.7 began to undergo recrystallization, with a weaker basal texture and a {0002} polar density of 3.163 m.r.d. Simultaneously, a main texture component <10$\bar{1}$0>//DD was also contained. This was closely related to the strong deformation texture of the G4.7 wire before annealing, which was a strong <10$\bar{1}$0> fiber texture, as detailed in our previous paper [11]. With the process of SRX (375, 400 °C), the basal texture component was markedly strengthened. Continuously heating up to 475 °C, the recrystallized grains significantly grew, accompanied by a more pronounced {0002} basal texture, with a strength of 11.635 m.r.d; that is, as the annealing temperature increased and recrystallization proceeded, the basal texture of the G4.7 was gradually enhanced. Simultaneously, the texture component <10$\bar{1}$0>//DD was gradually weakened. Overall, the texture of the as-annealed wire was relatively weak and the orientation distribution of the grains was relatively random at 375 °C/30 min, which was beneficial for the coordination of the plastic deformation.

It is worth noting that the G4.7 wire was still affected by the cold-drawn deformation texture and could not fully recover to the as-extruded texture even under high-temperature annealing. When the G4.7 wires were annealed, new crystal nuclei began to form in the deformed microstructure. Nevertheless, at this time, the proportion of low-angle grain boundaries was relatively large. Therefore, the annealing texture was greatly affected by the deformation texture in the early stage of SRX. As the annealing temperature increased, the recrystallized grains of the G4.7 wire began to grow, and the high-angle grain boundaries gradually replaced the low-angle grain boundaries of the microstructure. This is also a common phenomenon in the Mg alloy recrystallization process [26]. At this point, the formation of new grains could not follow the orientation of the deformed grains. Because the interface energy of grain boundaries with the same orientation was the smallest, it was not conducive to the formation and growth of new grain boundaries.

In addition, it was found that the texture intensity in the G4.7 wire gradually increased and the basal texture became more pronounced with the increase in annealing temperature. This is consistent with what has been reported in the literature [16]. The report states that the maximum texture intensity of as-rolled Mg-2.9 wt.%Zn alloy showed a significant change after annealing at 275 °C. When the annealing time was 5 min, the texture intensity markedly decreased, from 3.4 to 2.3, whereas the texture density began to increase as the annealing time was extended. After annealing for 60 min, the texture strength rose to 3.6, which was even greater than the deformation texture. What is more, the literature [16] suggested that {0002} polar intensity increased with the increase in grain size, mainly because the large grains engulfed the small grains, resulting in a decrease in texture components and a more concentrated orientation. The phenomenon of texture strengthening caused by grain growth was independent of Mg alloy composition. Therefore, the basal texture intensity of as-annealed G4.7 increased as the grains grew. A similar trend was also found in the AZ61 alloy: the texture continuously strengthened and finally formed a strong basal texture with a coarsening of the grains [27].

The texture remained after annealing, which reduced the cold drawing performance of the G4.7 wire. Based on the above results, it is believed that the nearly single-phase microstructure, which was more uniform and had regular grains, as well as a weaker annealing texture, contributed to the preparation of Mg alloy wires through cold drawing followed by annealing treatment.

## 5. Conclusions

To sum up, we performed high-temperature annealing on as-drawn G4.7 wire. The microstructural evolution and the textural evolution, as well as their effects on the subsequent plastic deformability of G4.7 wire, were investigated. It was found that the uniformity and regularity of the recrystallized grains, as well as the annealing texture, impact the follow-up cold drawing performance. With the increase in the annealing temperature, the basal texture was gradually strengthened. The inverse pole figure showed that the texture component transformed from <10$\bar{1}$0>//DD to a recrystallized texture based on <11$\bar{2}$0>//DD. Even under high-temperature annealing, the G4.7 wire was still affected by the cold-drawn deformation texture and could not fully recover to the as-extruded texture. Annealing at 375 °C/30 min caused the most uniform grain size and orientation distribution in the microstructures and thus contributed to a good subsequent drawing deformability with a cold drawing ATS of 144%. It is expected that more uniform and regular recrystallized grains and a more random distributed crystal orientation will be beneficial for the plastic deformation of Mg alloy wires.

## Figures and Tables

**Table 1:** The statistical results of as-annealed G4.7 alloy wires and corresponding ultimate accumulative drawing true strain.

Annealing Parameters	Statistical Results of Measured Grain Intercepts			Ultimate Accumulative Drawing True Strain after Annealing/%
	Mean /μm	Standard Deviation SD	Coefficient of Variation CV	
350 °C/30 min	3.8	1.33	0.35	131
375 °C/30 min	9.4	3.20	0.34	144
400 °C/30 min	18.8	8.22	0.44	144

## Data Availability

Data are contained within the article.
